# Supplementary material for: Depletion of Fumarate Hydratase, an Essential TCA Cycle Enzyme, Drives Proliferation in a Two-Step Model
Source: Cancers (Basel). 2022 Nov 9;14(22):5508. doi: 10.3390/cancers14225508 (PMC9688661; doi:10.3390/cancers14225508)
Supplement: Supplementary file 1 [file cancers-14-05508-s001.zip › cancers-1971203-supplementary.pdf]

cancers

Supplementary Materials: Depletion of Fumarate Hydratase, an Essential TCA Cycle Enzyme, Drives Proliferation in a Two-Step Model

Balakrishnan Solaimuthu, Michal Lichtenstein, Arata Hayashi, Anees Khatib, Inbar Plaschkes, Yuval Nevo, Mayur Tanna, Ophry Pines and Yoav D. Shaul

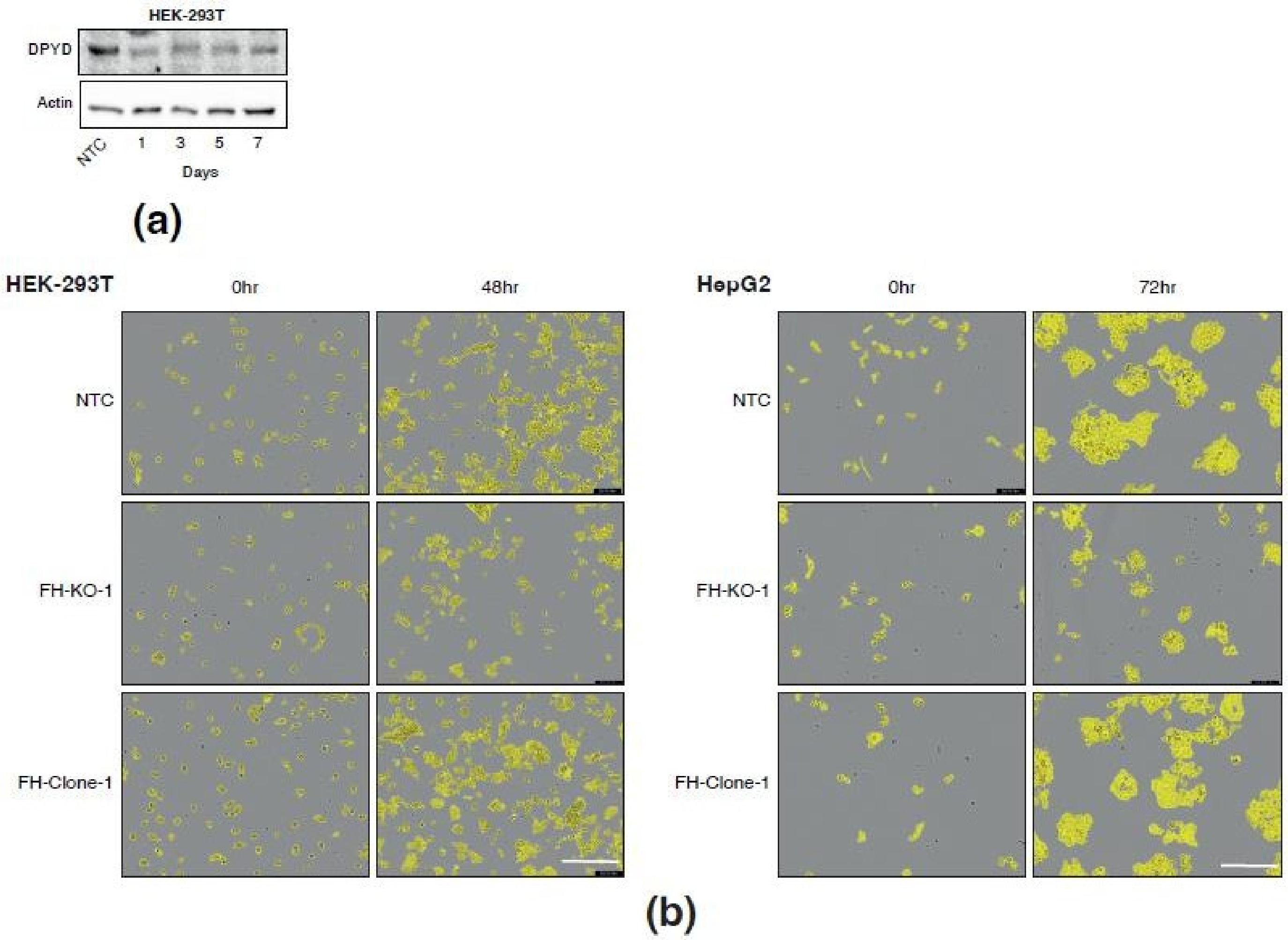

Figure S1. Short-term FH loss inhibits cell proliferation: (**a**) DPYD was silenced in HEK-293T using the CRISPR-Cas9 system and then subjected to 24 hours of puromycin selection. At the indicated timespost-selection (short-term), the cells were lysed and subjected to immunoblotting using the indi- cated antibodies. NTC is a nontargeted control. (**b**) Representative pictures of the proliferation assayas taken by the IncuCyte. Images representing the number of cells during 0 and 48 hours in HEK-293T and 0 and 70 hours in HepG2. Bar = 400 µm.

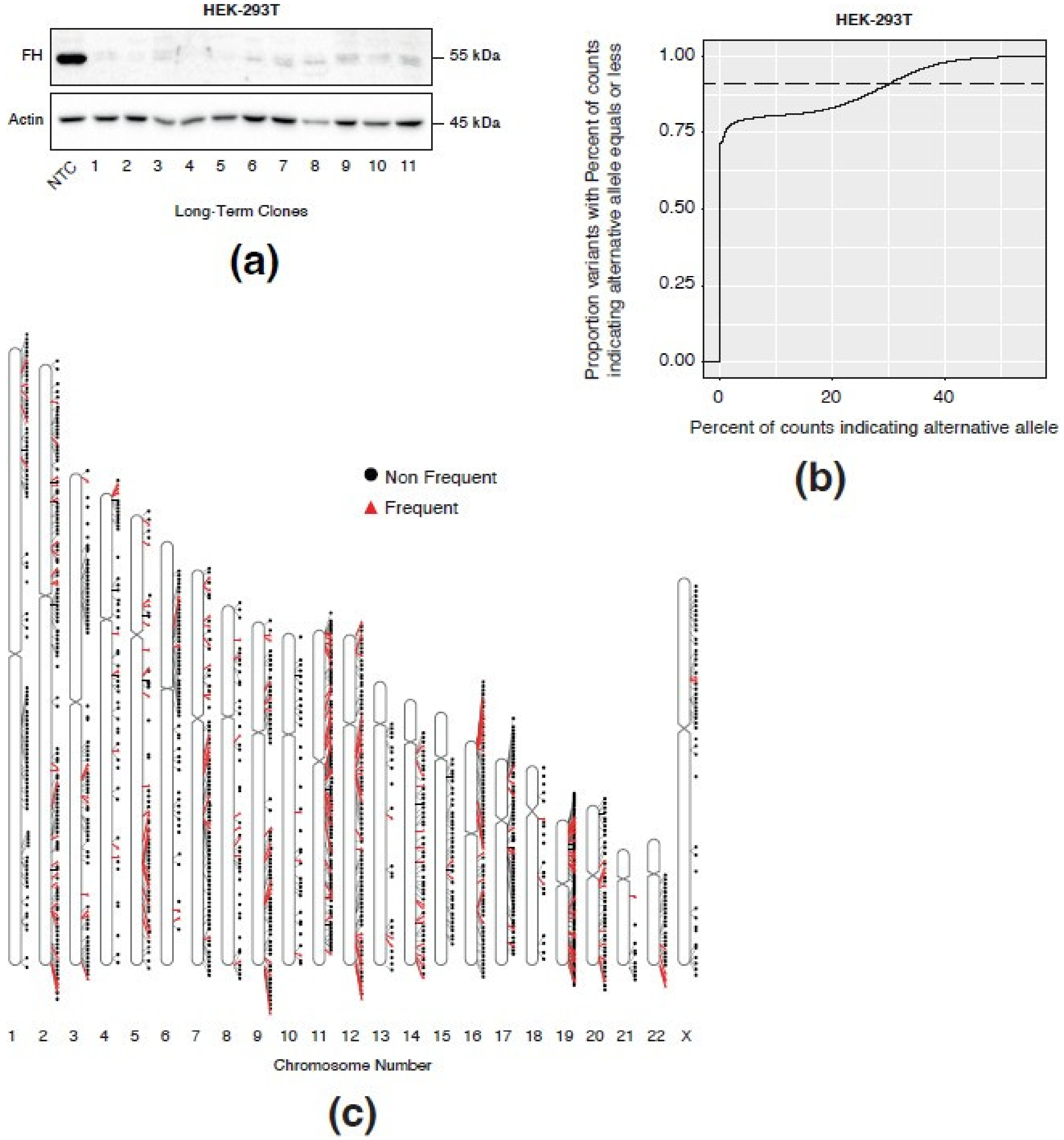

Figure S2. Distribution of the different variants: (**a**) Western blot indicating FH level in the 11 differentFH-KO long-term stable clones. The cells were lysed and subjected to immunoblotting using the indicated antibodies. NTC is a nontargeted control. (**b**) The cumulative distribution plot of the percent of counts indicates an alternative allele in the HEK-293T cell line. (**c**) The synonymous mutation distribution pattern is represented in the different chromosomes. The mutation that occurred in more than 4 clones, is considered a frequent mutation and marked as a red triangle.

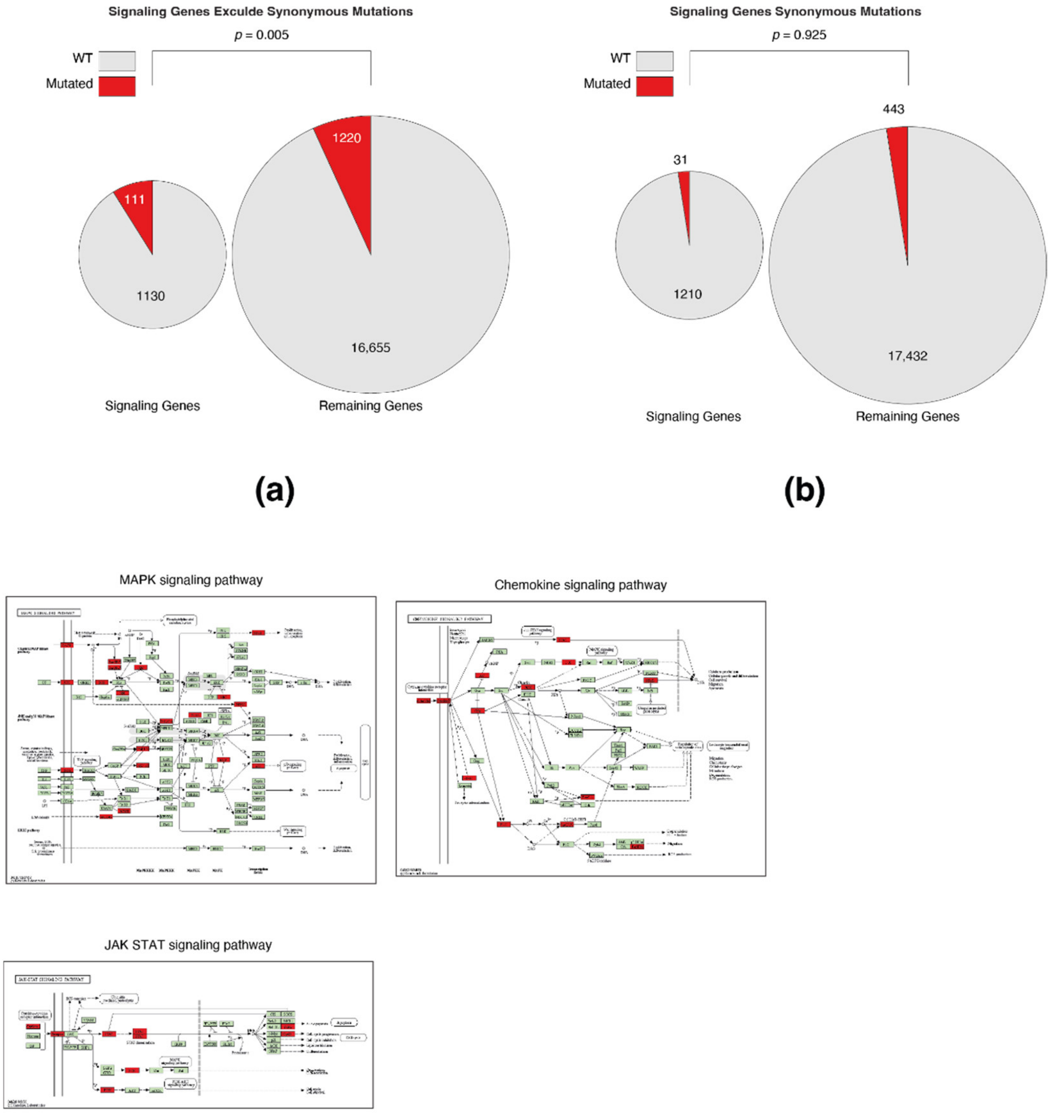

(c)

Figure S3. FH loss-dependent mutations are enriched in signaling genes: (**a**) Genes encoding for signalingmolecules are enriched with mutations caused by FH knockout. All of the signaling genes were defined based on the KEGG pathway database (https://www.genome.jp/kegg/pathway.html). The number of mutations (excluding synonymous) in the signaling and non-signaling genes were de- termined. A pie chart representing the proportion of mutations in the signaling genes versus the remaining genes. Pvalue is calculated by Fisher's exact test using R. (**b**) Same analysis as A but for synonymous mutations. (**c**) A selected set of genes found to be mutated in FH long-term clones are highlighted in the corresponding signaling cascade. Specifically, the genes were labeled using the KEGG color website (https://www.genome.jp/kegg/pathway.html) [8], which displays the distribution of the mutated genes (marked in red) in the different signaling pathways.

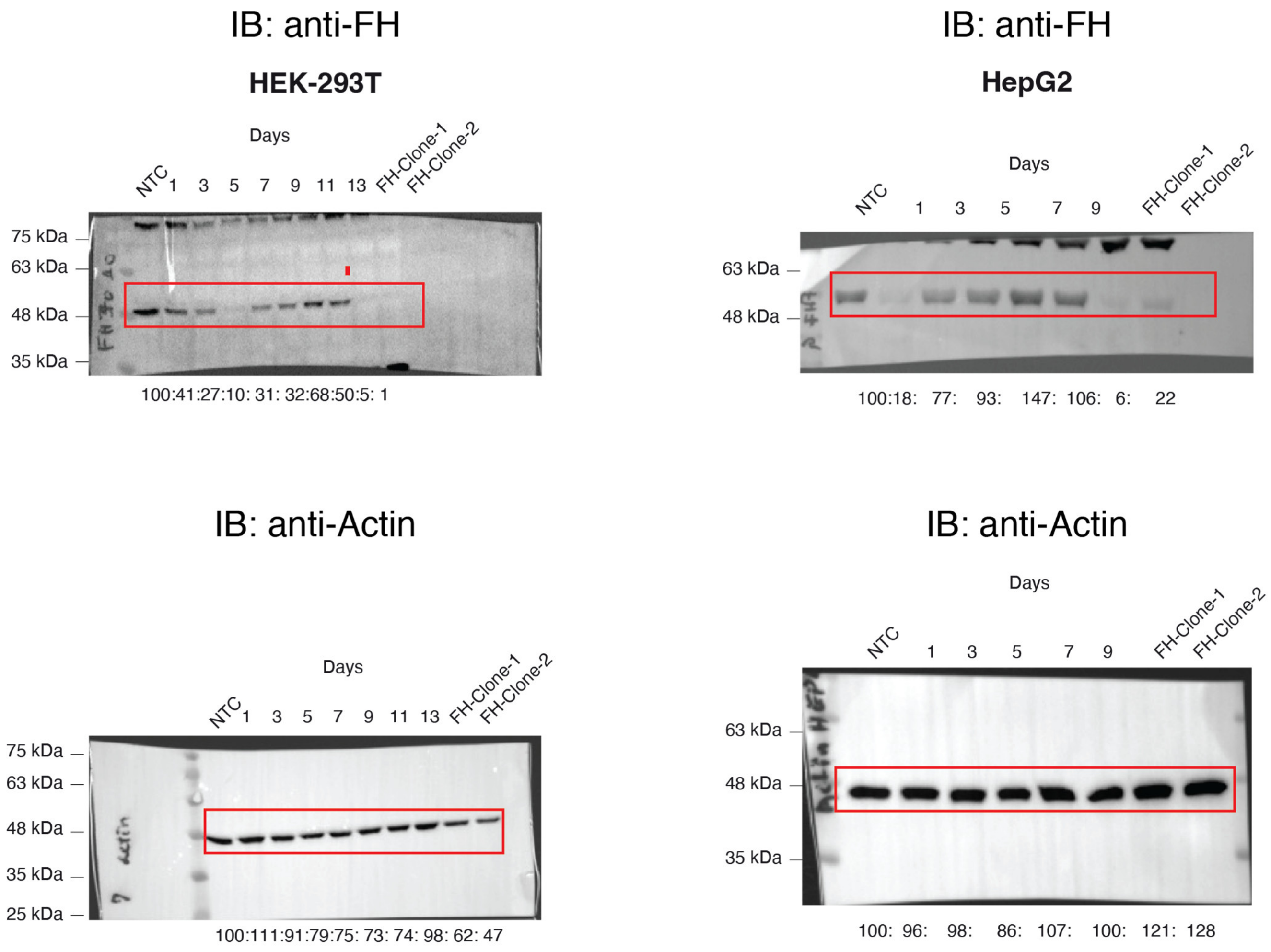

Figure S4. Uncropped Western Blots of blots shown in Figure 1.

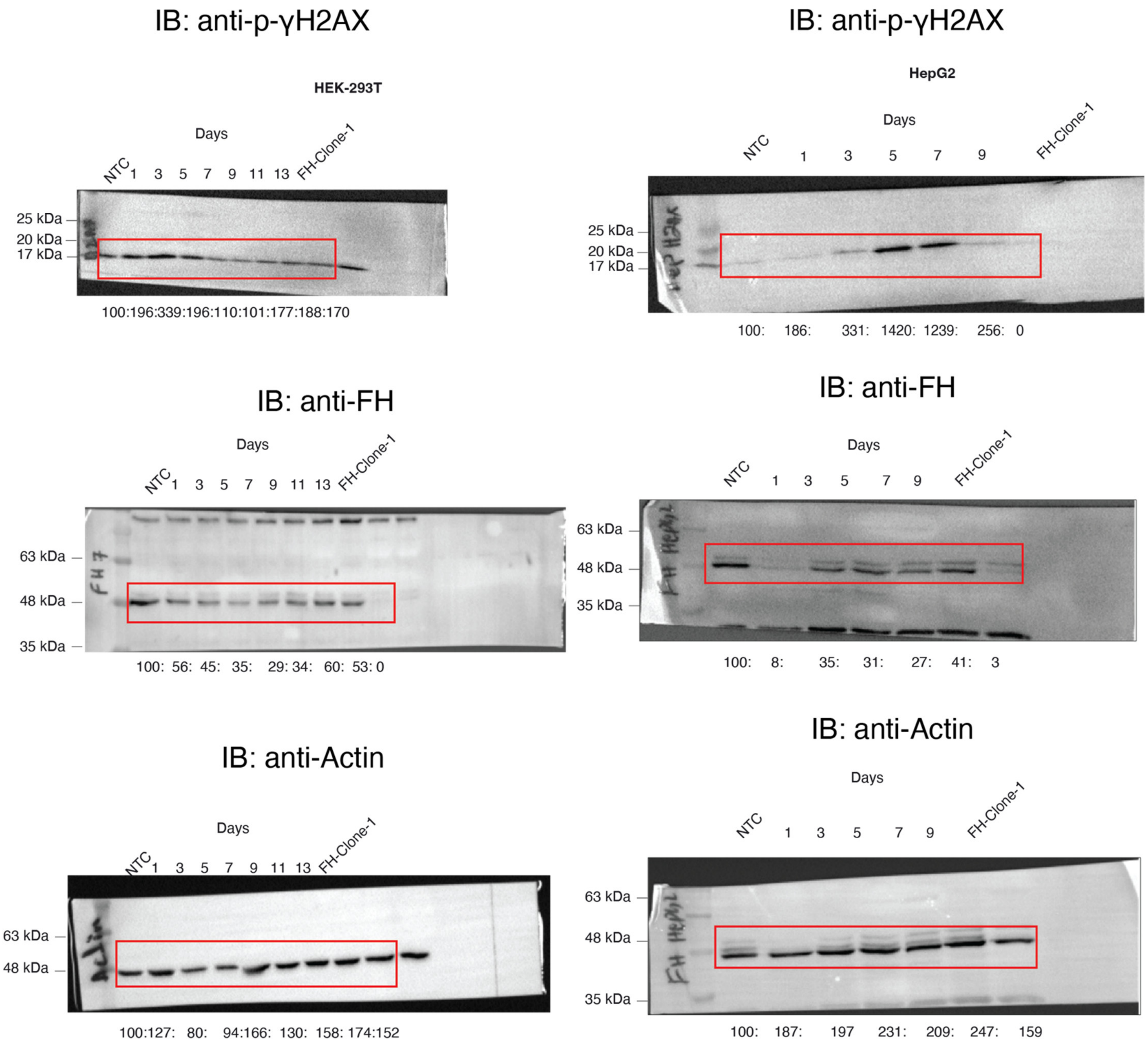

Figure S5. Uncropped Western Blots of blots shown in Figure 2.

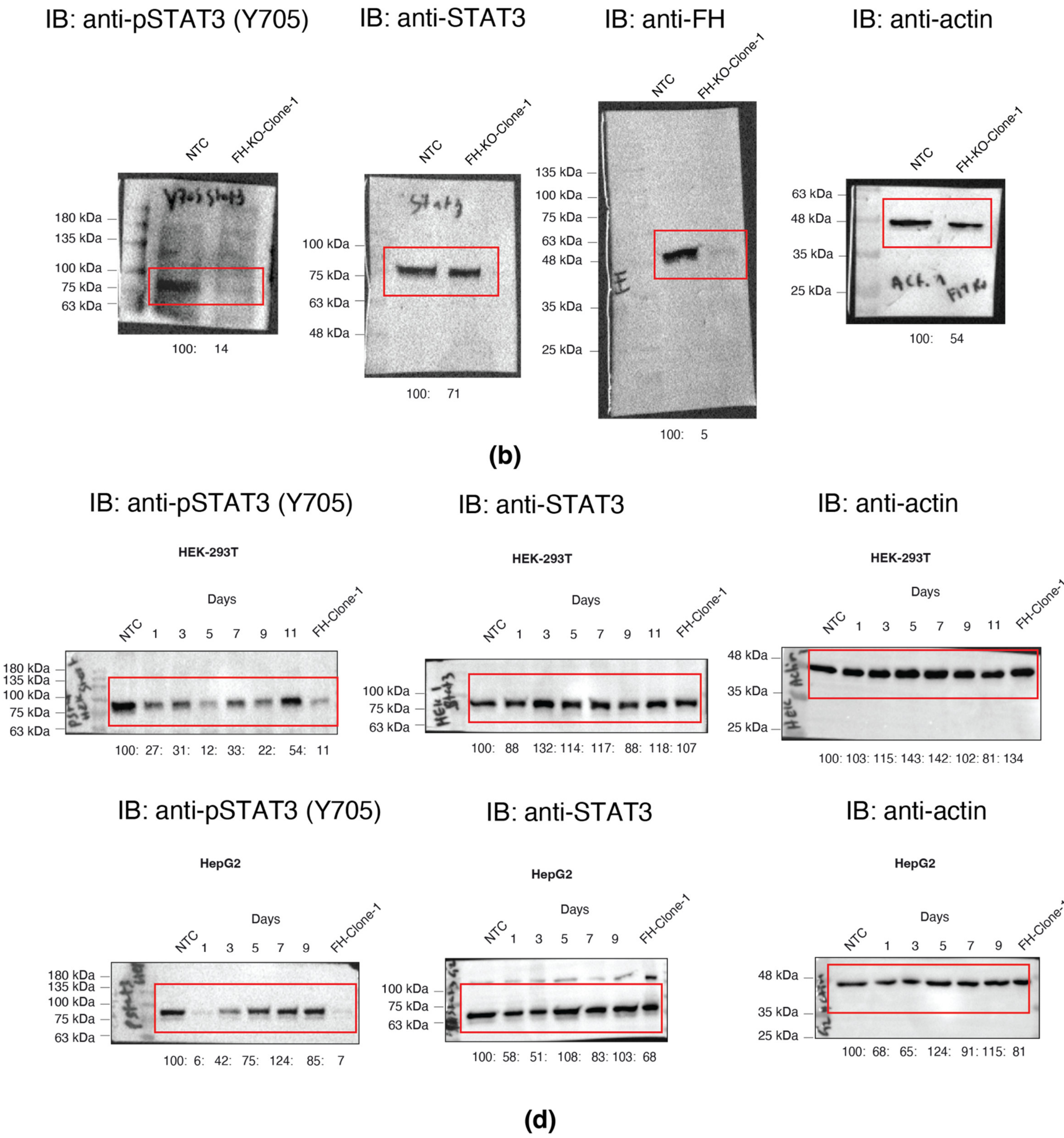

Figure S6. Uncropped Western Blots of blots shown in Figure 5.

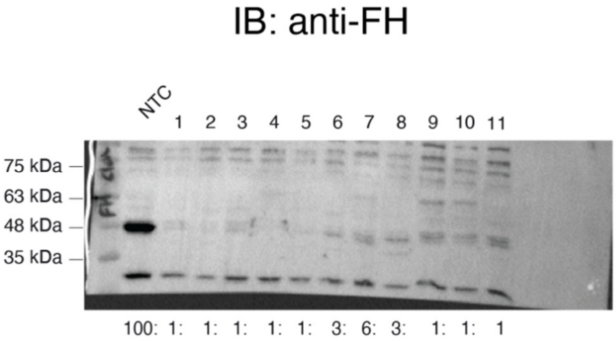

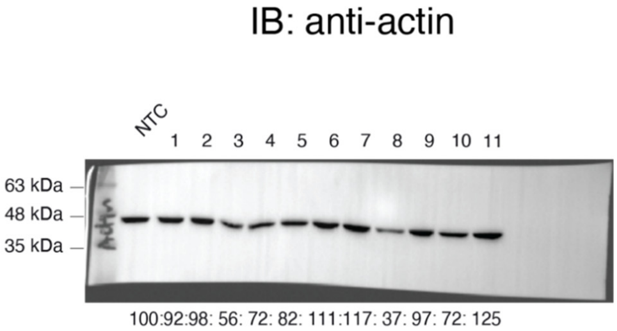

Figure S7. Uncropped Western Blots of blots shown in Figure S1 and S2.
